# Society Proceedings

**Published:** 1912-08

**Authors:** 


### SOCIETY PROCEEDINGS

---

THE 14TH ANNUAL MEETING of the *American Hospital As-* agement, philanthropy, nursing, etc., will be discussed. Dr. to Friday, Sept. 24—27, 1912. Various phases of business management, philanthorpy, nursing, etc., will be discussed. Dr. R. R. Ross of the Buffalo General Hospital will read on the Use of Salvarsan. The Secretary is J. N. E. Brown, M. B., 90 Charles St., East, Toronto, Ont.

---

The 13th Annaul Meeting of the Lake Keuka Medical and Surgical Association was held at Grove Springs, Lake Keuka, N. Y., July 11th and 12th, 1912.

Program:

Ectopic Gestation with a Case, J. F. Myers, Sodus.

Flatulence, John R. Williams, Rochester.

Diagnosis and Treatment of Perforated Duodinal Ulcer, Nathan Jacobson, Syracuse.

A Few Suggestions Regarding the Cure of Hernia, W. B. DeGarmo, New York City.

The Relation of Migraine to Autointoxication, Albert C. Snell, Rochester.

Crotalin in the Treatment of Epilepsy and Nerve Disorders, James B. Woodruff, Rochester.

Operations for Diseases of the Gall Bladder, A. L. Beahan, Canandaigua.

Salvarsan Versus Mercury, E. Wood Ruggles, Rochester.

Syphilis of the Nervous System with Especial Reference to the Use of Salvarsan, Floyd Crego, Buffalo.

Some Observations on New Methods of Diagnosis of Syphilis, Tom A. Groat, Syracuse.

Diagnosis in Brain Injuries, Herbert A. Smith, Buffalo.

Surgical Procedures in Brain Injuries, Frank W. McGuire, Buffalo.

The Advantages of the Laboratory to the Country Physician, E. C. Foster, Penn Yan.

Epidemic Pneumonia, L. C. Lewis, Belmont.

Clinical Teaching in the Fifth College year, John L. Heffron, Syracuse.

Typhoid Vaccine as a Prophyolatic and Therapeutic Measure, A. E. Larkin, Syracuse.

---

The fourteenth annual meeting of the American Proctologic Assocation was held at Atlantic City, N. J., June 3 and 4, 1912, the president, Dr. John L. Jelks, of Memphis, Tenn., in the chair.

Officers elected for the ensuing year: President, Louis J. Hirschman, M. D., Detroit, Mich.; Vice-President, Alois B. Graham, M. D., Indianapolis, Ind.; Secretary-Treasurer, Lewis H. Adler, Jr., M. D., Philadelphia, Pa. The place of meeting for 1913 will be at Minneapolis, Minn. Exact date and headquarters to be announced later.

The September issue of the *Proctologist* (St. Louis) will contain a full report of papers and discussions.

---

The fourth annual meeting of the American Association of Clinical Research will be held in New York City, at the Academy of Medicine, on November 9, 1912.

The sessions will be held from 9 a. m. to 1 p. m., from 3. p. m. to 6 p. m. and from 8 to 10 p. m. The evening session will be open to the public.

Notable contributions on the Negri Bodies, on certain Fluids for Tubercle Bacilli in the Urine, on Adjustment and Function, on Psychoanalysis and Traumbedeutung, on a Pandemic of Malignant Encapsulated Throat Coccus, on The Single Remedy on Indicanuria and Glycosuria, on Disease Conditions expressive of Correct Diagnosis, on Biochemic Problems, on The Two Most Far-Reaching Discoveries in Medicine, and others are to be given.

The permanent secretary, Dr. James Krauss of Boston, urges that local branches be formed in various large cities.

